# A novel channel selection method for optimal classification in different motor imagery BCI paradigms

**DOI:** 10.1186/s12938-015-0087-4

**Published:** 2015-10-21

**Authors:** Haijun Shan, Haojie Xu, Shanan Zhu, Bin He

**Affiliations:** College of Electrical Engineering, Zhejiang University, Hangzhou, 310027 China; Department of Biomedical Engineering and Institute for Engineering in Medicine, University of Minnesota, Minneapolis, MN 55455 USA

**Keywords:** EEG, Brain-computer interface (BCI), Motor imagery (MI), Relief, IterRelCen, Channel selection

## Abstract

**Background:**

For sensorimotor rhythms based brain-computer interface (BCI) systems, classification of different motor imageries (MIs) remains a crucial problem. An important aspect is how many scalp electrodes (channels) should be used in order to reach optimal performance classifying motor imaginations. While the previous researches on channel selection mainly focus on MI tasks paradigms without feedback, the present work aims to investigate the optimal channel selection in MI tasks paradigms with real-time feedback (two-class control and four-class control paradigms).

**Methods:**

In the present study, three datasets respectively recorded from MI tasks experiment, two-class control and four-class control experiments were analyzed offline. Multiple frequency-spatial synthesized features were comprehensively extracted from every channel, and a new enhanced method IterRelCen was proposed to perform channel selection. IterRelCen was constructed based on Relief algorithm, but was enhanced from two aspects: change of target sample selection strategy and adoption of the idea of iterative computation, and thus performed more robust in feature selection. Finally, a multiclass support vector machine was applied as the classifier. The least number of channels that yield the best classification accuracy were considered as the optimal channels. One-way ANOVA was employed to test the significance of performance improvement among using optimal channels, all the channels and three typical MI channels (C3, C4, Cz).

**Results:**

The results show that the proposed method outperformed other channel selection methods by achieving average classification accuracies of 85.2, 94.1, and 83.2 % for the three datasets, respectively. Moreover, the channel selection results reveal that the average numbers of optimal channels were significantly different among the three MI paradigms.

**Conclusions:**

It is demonstrated that IterRelCen has a strong ability for feature selection. In addition, the results have shown that the numbers of optimal channels in the three different motor imagery BCI paradigms are distinct. From a MI task paradigm, to a two-class control paradigm, and to a four-class control paradigm, the number of required channels for optimizing the classification accuracy increased. These findings may provide useful information to optimize EEG based BCI systems, and further improve the performance of noninvasive BCI.

## Background

Brain-computer interfacing is a technology which offers an alternative communication mode without going through the normal neuromuscular pathways [1–10]. It makes use of brain signals to convey communication and control information, and in general, noninvasive electroencephalogram (EEG) is a widely used modality to measure brain signals. Of all types of brain-computer interfaces (BCIs), we mainly focus on sensorimotor rhythm based BCI, which relies on imagination of movement of a limb or other parts of the body to induce EEG signals in corresponding brain areas [3, 10–12]. These signals can then be decoded and translated into control commands for specific output devices, e.g., cursor movement [13–15] or neuroprostheses [16].

One of the challenges in the development of an effective sensorimotor rhythm based BCI system is to discriminate amongst different motor imagery (MI) tasks, such as the imagination of movement of left hand, right hand, and feet. Generally speaking, classification of different motor imageries mainly depends on the following aspects: data preprocessing, extraction of subject-specific features, and appropriate classifiers. However, aside from these, there exists another important aspect that researchers typically ignore, and that is channel selection, i.e., selecting the least number of channels that yield the best accuracy. The use of additional channels was discovered to improve classification performance. However, this does not mean the more the better. Actually, a large set of channels without going through channel selection will include noisy and redundant channels, which would deteriorate the BCI system performance. Moreover, the use of more channels increases the cost of BCI system [17, 18]. Therefore, it is necessary to adopt an effective approach to select the optimal channels out of the full channel set. Several groups have investigated this question. For example, in [19], the authors showed that using the algorithms REF and 10-Opt based on SVM, and the number of channels can be significantly reduced without an increase of error. Such method mainly relies on a specific classifier to evaluate the feature set. In [20, 21], the CSP method and its extension SCSP were employed to conduct channel selection on MI datasets, showing that most of the channels can be removed. Other methods based on mutual information [22], GA-ANN [23], etc., separately showed it was a feasible way to reach optimal accuracy. But they either ignore the correlation between channels, or consume a long period of time to calculate. Relief [24] is a widely used feature selection method which is independent of classification algorithms, thus it is effective in computation. Based on its simple but effective algorithm principle, Relief has been proved to perform well in a large number of applications. However, due to its distance measure principle, Relief is highly sensitive to artifacts and noise, which would be included in multiple-channel EEG data. In this study, a novel iterative Relief based on distance from center (IterRelCen) algorithm was proposed for optimal channel selection. IterRelCen is an enhanced approach based on the principle of Relief/Relieff.

By now, the channel selection studies in most of the existing literatures were performed in datasets recorded from MI task paradigm (details about the paradigm described in the “Methods” section). Few studies were conducted in datasets recorded from control paradigms (two-class and four-class control paradigms, details about the paradigms described in the “Methods” section). Like the study in [25], it focused on research on online autocalibration and channel selection for adaptive BCI system. However, studies have shown that the characteristics of EEG signal recorded from control paradigms may be inherently different from the EEG data recorded from MI tasks paradigms. For example, it was demonstrated that an increase of task complexity or attention influences brain activities [26, 27]. In addition, the introduction of real-time visual feedback (only exists in control paradigms) would augment brain activity over motor areas [28]. Furthermore, it has been shown that the visual stimuli or visual motion stimuli (only exists in control paradigms) can evoke brain activities in different scalp distributions [29]. Based on these, we believe that the problem of channel selection in datasets recorded from control paradigms needs to be evaluated.

In the present study, the proposed method IterRelCen was used for optimal channel selection over three different datasets, respectively recorded from MI tasks paradigm, two-class control paradigm and four-class control paradigm. It was found that due to the paradigm difference, it requires different number of EEG channels to reach optimal performance in these datasets, and the control paradigms necessitate more channels than MI tasks paradigm.

## Methods

### Experiment and data description

In this study, three datasets were used. The specific information about each dataset and its corresponding experimental paradigm are described as follows:

1. *Dataset 1* from a MI task paradigm. The dataset was made available by Dr Allen Osman of the University of Pennsylvania [30] for a data analysis competition during the Neural Information Processing System (NIPS2001) [31]. In this experimental paradigm, subjects were asked to imagine left or right hand movement once the letter “L” or “R” was shown on a computer screen. Subjects then performed sustained hand movement imagery in a fixed period of time. The specific experimental process is illustrated in Fig. 1a. Data from eight subjects were used in this study, and the scalp EEG was recorded from 59 channels (international 10–20 system) with a sampling rate of 100 Hz. For each subject, the total number of trials is 180:90 trials for left and 90 trials for right. The length of each trial is consistent, 2.25 s.

**Fig. 1:**
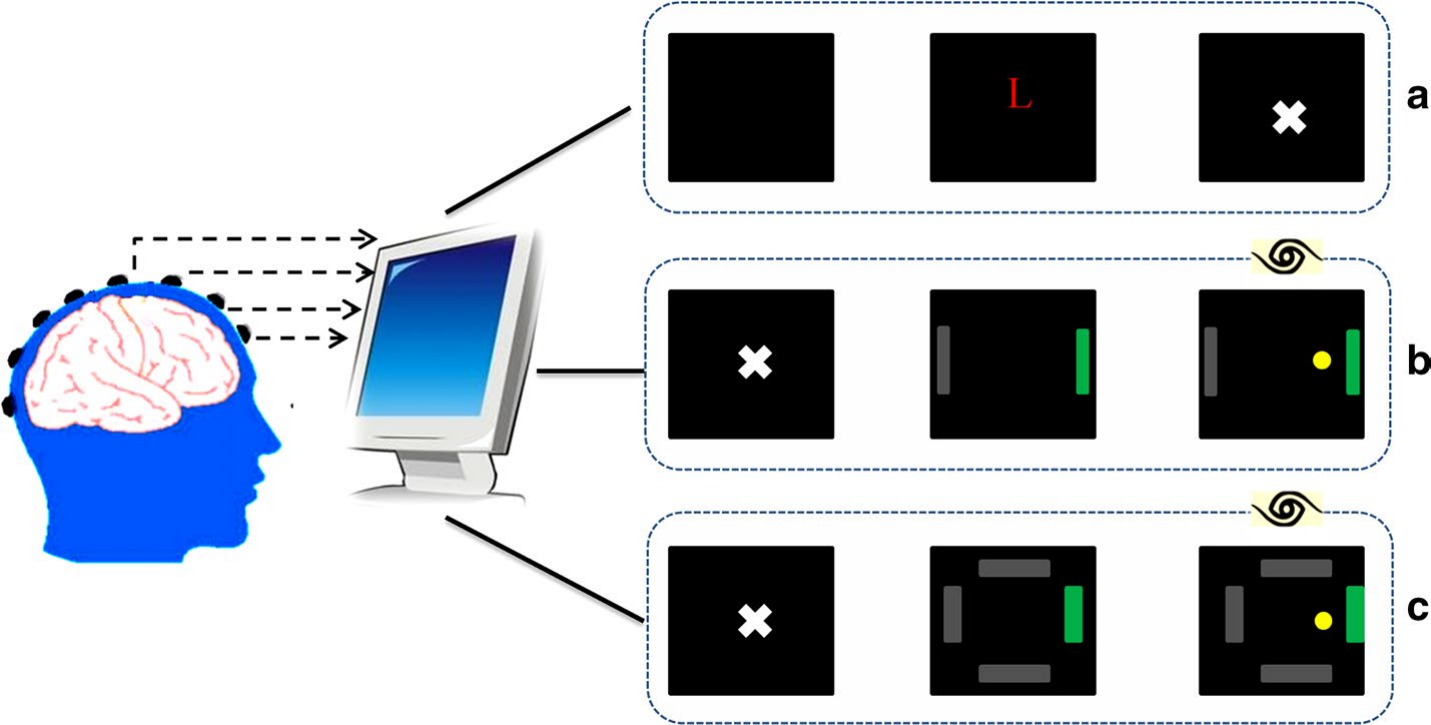
Illustration of three different MI paradigms. This diagram is presented here to provide a general knowledge of the difference among the three MI paradigms. **a** MI tasks paradigm; **b** two-class control paradigm (also named 1-D cursor control); **c** four-class control paradigm (also named 2-D cursor control). Three experimental stages presented in each paradigm are rest stage, preparation stage and execution stage. In the execution stage of paradigms **b** and **c**, eyes are open, with real-time visual feedback; whereas in execution stage of paradigm **a**, eyes are closed without visual feedback
2. *Dataset 2* from a two-class control paradigm. The experiments were conducted in the Biomedical Functional Imaging and Neuroengineering Laboratory at the University of Minnesota according to a protocol approved by the Institutional Review Board of the University of Minnesota [13]. In this experimental paradigm (Fig. 1b), at the beginning, the screen was blank. Two seconds later, a target appeared at one of two locations on the periphery of the screen. At 5 s, the cursor appeared in the center of the screen and subjects can move the cursor horizontally through imagination of left or right hand movement. Provided with visual feedback of the cursor position, subjects could make real-time adjustment of their imagination patterns to control the cursor movement, and this is the major difference with the MI task paradigm. A trial is finished if the cursor reached the target bar within 6 s, reached the wrong target, or failed to reach the target within 6 s. So each trial lasts 5–11 s. However, not all the time points of a trial carry information about the EEG modulation of motor imageries, so only the execution period of each trial was extracted for analysis, usually 0.5–3 s. Data from eight subjects were used, and the scalp EEG was recorded from 62 channels (international 10–20 system) with a sampling rate of 200 Hz. For each subject, the total number of trials is 180:90 trials for left and 90 trials for right.
3. *Dataset 3* from a four-class control paradigm. The experiments were also conducted in the Biomedical Functional Imaging and Neuroengineering Laboratory at the University of Minnesota according to a protocol approved by the Institutional Review Board of the University of Minnesota. This paradigm is similar with the two-class control paradigm. The only difference is that the four-class control paradigm controls the movement of a cursor in four directions, i.e., MI of left hand, right hand, both hands, and nothing control cursor move to left, right, up, and down. The experimental process is illustrated in Fig. 1c. Each trial lasts 5–11 s, and the execution period of each trial was extracted for analysis, usually 0.5–3 s (same with Dataset 2). Data from eight subjects were used here, and the scalp EEG was recorded from 62 channels (international 10–20 system) with a sampling rate of 1000 Hz. For each subject, the total number of trials is 280:70 trials for each direction.

Note that there is a little difference in electrode setups between Dataset 1 and Dataset 2/3 (The electrode setup of Dataset 3 is the same with Dataset 2). Only three peripheral electrodes were not recorded during the Dataset 1 experiment. The other electrode locations were consistent with Dataset 2, so this does not affect the results.

### Data processing

Dataset 3 had a relatively high sampling rate, so before signal processing, we down sampled the EEG data from Dataset 3 from 1000 to 200 Hz. In order to be consistent with the sampling rate of Dataset 1, Dataset 2 and Dataset 3 were also down sampled to 100 Hz; we found that whether the sampling rate was consistent or not, it did not affect the result. So Dataset 2 and Dataset 3 were only down sampled to 200 Hz for the results presented here. Then, the data processing was carried out in the following steps: spatial filtering, feature extraction and feature normalization.

#### Surface Laplacian filtering

Raw scalp EEG has a low signal-to-noise ratio, since it is spatially smeared due to the head volume conductor effect. Thus, in order to improve the quality of EEG, a surface Laplacian filter [32] was adopted to accentuate localized activity and to reduce diffusion in the multichannel EEG [33, 34]. The formulation for the surface Laplacian filter is as follows:

1 $$V_{j}^{Lap} = V_{j} - {1 / n}\sum\limits_{{k \in s_{j} }} {V_{k} }$$

where *V*_*j*_ represents the target channel, *V*_*k*_ stands for neighboring channels, and *S*_*j*_ is the index set of the surrounding channels. Parameter *n* is the number of neighboring channels, and in most cases, *n* is set to 4, whereas *n* is 2 or 3 when the target channel is at the periphery.

#### Feature extraction

A frequency range from 5 to 35 Hz was focused on, since many studies have indicated that this range mainly represents the intent of user in MI-based BCI. Furthermore, the phenomenon of event-related desynchronization (ERD) and synchronization (ERS) in the mu band (8–12 Hz) and beta band (18–26 Hz) [11, 13, 35] occuring during motor imagination over sensorimotor cortex are also included in this frequency range. Considering that the related frequency bands are narrow banded, we decomposed the entire 5–35 Hz into 13 partially overlapping sub-bands [36], using constant-Q scheme, which is also known as the proportional band width. According to the computing principle, the 13 sub-bands are 5.25–6.75 Hz, 6.0–7.71 Hz, 6.86–8.82 Hz, 7.84–10.08 Hz, 8.96–11.52 Hz, 10.24–13.16 Hz, 11.70–15.04 Hz, 13.37–17.19 Hz, 15.28–19.64 Hz, 17.46–22.45 Hz, 19.96–25.66 Hz, 22.81–29.32 Hz, and 26.07–33.51 Hz, respectively. We were mainly concerned about the power changes of these frequency bands, and therefore we extracted the envelope of each sub-band using the Hilbert transform, given that the envelope could quantitatively reflect the instantaneous power change of a frequency band. In this study, each envelope was extracted from a trial data. Each feature is the average of single envelope over trial time.

#### Feature normalization

Min–max normalization was adopted here to transform feature values into the range (−1, 1). The normalization was performed according to the formula below.

2 $$XN = (newMax - newMin)\frac{X - Min}{Max - Min} + newMin$$

where *Min* and *Max* are the minimum and maximum values of a feature. The parameters *newMin* and *newMax* are the low and upper bound of new range, and *X* is the feature value that needs to be transformed.

Through the above process, we could obtain 13 normalized features during a trial from a single channel, and the 13 features corresponded to the aforementioned 13 sub-bands. When all the channels (59 or 62) were initially used, the total number of features would be 767 or 806 (13 × 59/62). During the computing process, features from different channels are concatenated into a feature vector representing one trial.

### Channel selection

#### Performance assessment

Channel selection is an optimization problem which chooses the optimal subset of channels from the full set available. Our ultimate goal is to maximize the classification accuracy of distinct motor imageries in MI-based BCIs, so optimal here means that achieving best accuracy by using least number of channels. Here, the best accuracy means the optimal testing accuracy in classification, which is obtained by computing the mean value of testing accuracy of each fold in 10-fold cross validations.

#### Relief and Relieff

**Fig. 2:**
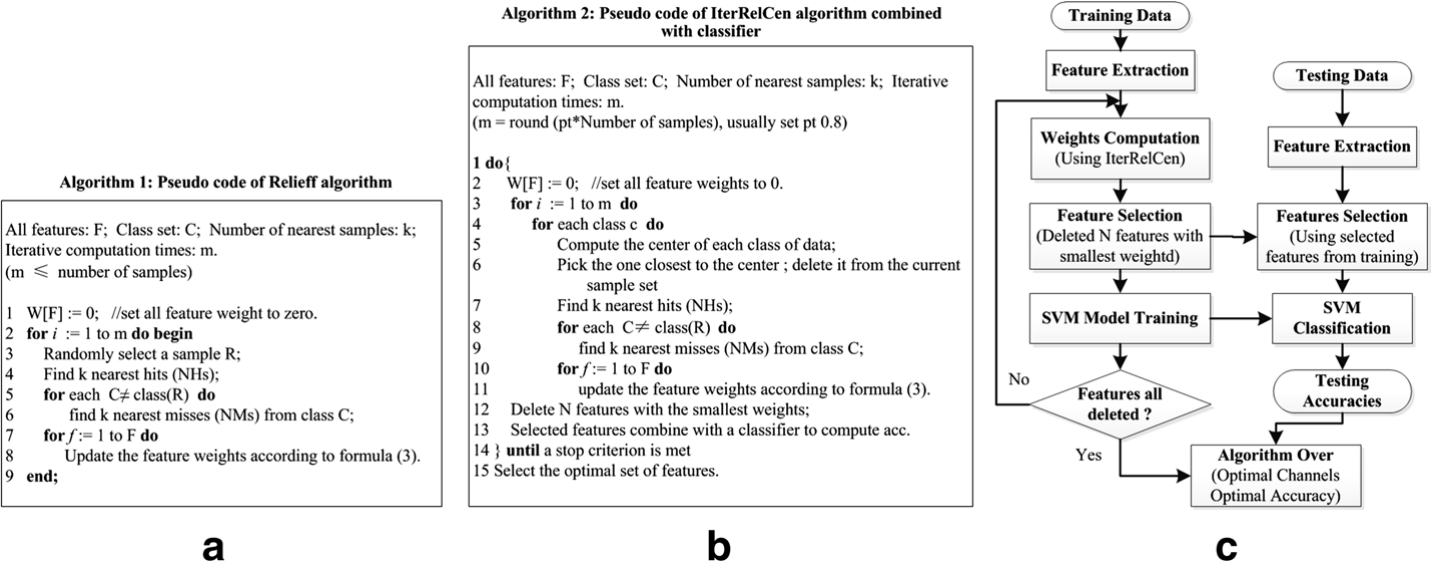
Pseudo code of Relieff and IterRelCen algorithms and flow chart of the whole method. **a** Pseudo code of Relieff algorithm; **b** pseudo code of IterRelCen algorithm; **c** the flow chart of the whole method for channel selection

Relief [24] is a widely used method for feature selection in binary classification problems, due to its effectiveness and simplicity of computation. Relieff [37] is an extension of it, designed for feature selection in multi-class classification problems. Relief and Relieff are similar in algorithm principle. So here we only take Relieff as an example to describe the computing principle. For technical details about Relief, please refer to [24]. A key idea of Relieff is to estimate the quality of attributes according to their abilities of distinguishing among samples that are near to each other, given in Fig. 2a. At the beginning, the weights for all the features are initialized to zeros (line 1). Then it randomly selects a sample *R* from the training dataset *T* (line 3), and selects the nearest *k* samples from the same class training set (called “Near Hits”) (line 4) and nearest *k* samples from each of the other classes (called “Near Misses”) (lines 5 and 6). It updates the feature weight vector *W* for all attributes according to Eq. (3). The whole process would be repeated *m* times, where *m* is a user-defined parameter. Through the above described process, it could be easily understood that a large weight means the feature is important for the classification and a small one means less important. The weight computing equation is as follows:

3 $$\begin{aligned} W(f) & = W(f) - {{\sum\limits_{{j = 1}}^{k} {d(f,R,H_{j} )} } \mathord{\left/ {\vphantom {{\sum\limits_{{j = 1}}^{k} {d(f,R,H_{j} )} } {(m \cdot k)}}} \right. } {(m \cdot k)}} \\ & \quad + {{\sum\limits_{{C \ne class(R)}} {\left[ {\frac{{p(C)}}{{1 - p(class(R))}}\sum\limits_{{j = 1}}^{k} {d(f,R,M_{j} (C))} } \right]} } \mathord{\left/ {\vphantom {{\sum\limits_{{C \ne class(R)}} {\left[ {\frac{{p(C)}}{{1 - p(class(R))}}\sum\limits_{{j = 1}}^{k} {d(f,R,M_{j} (C))} } \right]} } {(m \cdot k)}}} \right. } {(m \cdot k)}} \\ \end{aligned}$$

where *W(f)* represents the weight of feature *f*. Parameter *k* is the number of nearest samples selected, and *m* represents the number of repeated times of computing process. *C* represents one class except for the *R*’s class. Parameter *p* is the prior probability of a certain class. Function *d(f, R, R*_*o*_*)* calculates the difference between sample vector *R* and sample vector *R*_*o*_ at the feature *f*, and *R*_*o*_ could be *H*_*j*_ (Near Hit) or *M*_*j*_ (Near Miss). For numerical attributes, *d(f, R, R*_*o*_*)* is defined as:

4 $$d ( {f,R,R_{o} } )= \frac{{\left| {value( {f,R}) - value ( {f,R_{o} } ) } \right|}}{{\hbox{max} ( f ) - \hbox{min} ( f )}}$$

*IterRelCen*. In Relief (Relieff) algorithm, the weight computation is affected by selection of target sample and selection of nearest neighbors (samples). However, these two aspects may provide a chance to bring in errors in weight computation. Specifically, (1) target sample is randomly chosen from the training dataset. Such strategy gives noisy samples (particularly the samples far away from the center of sample data in the same class) a chance to be selected. And it is undoubted that the use of noisy samples would easily bring in bias in weight computation. (2) The selection of nearest neighbors depends on the distances away from target sample, however the distance computation is determined by the features participated in. In our EEG dataset, the samples are with high-dimension features which could be mixed with noises or redundant features. Certainly such features would interfere with the distance computation between samples, and cause an error in feature weight computation. To solve the above two mentioned problems, an enhanced method “IterRelCen” based on the principle of Relief (Relieff) was proposed in this paper, given in Fig. 2b. The method IterRelCen reconstructed Relief (Relieff) algorithm from two aspects: First, the target sample selection rule is adjusted. Instead of randomly selecting sample, samples close to the center of dataset from the same class have the priority of being selected first (lines 5 and 6) according to formula (5), given that such samples could discriminate different class samples more accurately. Second, the idea of iterative computation is borrowed to eliminate the noisy features in samples (lines 1 and 14). In each iteration, the N features with the smallest weights (N is a user-defined parameter, depending on the required iterative speed) are removed from the current feature set (line 12). The left features are fed into the classifier for accuracy calculation (line 13). Repeat this process until the current feature set is empty. Usually, first removed ones are the features performing worst in discrimination. With the noisy features being gradually removed, the distance between samples computed from the rest features will reflect the relationship between samples more and more accurately.

5 $$\hbox{min} Dist=\left| {S_{i} - Ct} \right|,\forall S_{i} , \,\,where\,\,Ct = {1 \mathord{\left/ {\vphantom {1 n}} \right. } n}\sum\limits_{1}^{n} {S_{i} }$$

### Classification algorithm

A support vector machine (SVM) was used as the classification algorithm in this study. Classical SVM is a technique developed previously [38] to solve the two-class classification problem. The main idea of this typical SVM is to separate a two-class dataset by finding the maximum geometrical margin between the two classes.Given a training set of instance-label pairs $(X_{i} , y_{i} ),\, i = 1,\ldots ,l$, where $X_{i} \in R^{n}$ and $y_{i} \in \{ 1,{- 1} \}$. In order to build the SVM model, the following optimization problem needs to be solved:

6 $$\begin{aligned} \mathop {\hbox{min} }\limits_{\omega ,b,\xi } \frac{1}{2}w^{T} w + C\sum\limits_{i = 1}^{l} {\xi_{i} } \hfill \\ subject\,to\,\,y_{i} (w^{T} \phi (x_{i} ) + b) \ge 1 - \xi_{i} , \hfill \\ \xi_{i} \ge 0, i = 1, \ldots , l \hfill \\ \end{aligned}$$

The slack variables $\xi_{i}$ are introduced in case a small part of the data is nonlinearly separable. The margin is defined as $\gamma = 1/2 \| w \|$, so our goal is to make a best trade-off between low training error $\sum {\xi_{i} }$ and large margin $\gamma$. We also adopted a kernel function $\phi (X_{i} )$ to map training vectors $X_{i}$ onto a higher dimensional space. Thus, combined with slack variables, most of the nonlinear problems can be transformed into linear problems. There are several kernel functions provided to be chosen, e.g. linear function, polynomial function or radial basis function (RBF). Here, RBF was used.

Classical SVM only has the ability to discriminate between two classes; however, one of our datasets was from four-class control paradigm. Thus, we implemented the “one-against-one” approach [39] here to solve this multiclass classification problem. If *k* is the number of classes, then $k( k - 1)/2$ classifiers need to be constructed and each classifier model will then be trained from two-class data.

As shown in Fig. 2c, the channel selection process binds the channel selection method (IterRelCen) with classification algorithm. The generalization accuracies were estimated by ten-fold cross validation (tenfold CV) [40], in which a whole dataset is split up into ten folds. In each fold, feature selection using IterRelCen is performed first based on training set, resulting in a specific ranking of all features. And then it employs selected features to train the SVM model. Finally SVM is tested on the corresponding features of the test set to evaluate the testing accuracy. The classification accuracy was the average of testing accuracies of each fold, denoted by the following equation:

7 $$Acc = \frac{1}{k} \sum\limits_{i = 1}^{k} {acc_{i} } , \,\, where\, acc_{i} = \frac{{Correct\, Num_{test} }}{{Total\, Num_{test} }}$$

where *k* is 10, and *acc*_*i*_ is the testing accuracy of one fold.

Through the above method, the feature subset that achieved highest accuracy is recognized as optimal features. And channels hold at least one feature are considered as optimal channels. Note that by such method some optimal channels may only include several feature bands (less than 13), while some may retain all the 13 feature bands.

## Results

### Classification and channel selection results

In this study, three datasets from different experimental paradigms were analyzed. The channel selection was initiated with all the channels, e.g., 59 channels for MI tasks paradigm and 62 channels for two-class control and four-class control paradigms. The proposed IterRelCen algorithm was used to reduce the redundant features and select informative channels.

Table 1 summarizes the performance (optimal classification accuracies) of all the subjects from the three datasets. According to the results, the proposed method in this study yielded average classification accuracies of 85.2, 94.1, and 83.2 %, respectively for Dataset 1, Dataset 2, and Dataset 3. Compared to offline analysis results, the online average performances in Dataset 2 and Dataset 3 were only 84.9 and 70.8 %, respectively. The optimal results were compared with the results of using all the channels and the channel combination of C3, C4 and Cz (three typical motor imagery channels) in each dataset. Compared with using all the channels, the average accuracies were improved by 31.7, 8.0 and 19.7 % respectively for dataset 1, dataset 2 and dataset 3. And compared with using (C3, C4, Cz) channels, the average accuracies were improved by 22.9, 23.5 and 18.7 %. It can be concluded that the performance was greatly improved through channel selection. The p-values shown in the last row of Table 1 were obtained from the one-way ANOVA among the results of optimal channels, all the channels and three typical channels (C3, C4, Cz) in each dataset. It is observed that all the p-values in each dataset are below 0.05, indicating that the improvement caused by channel selection is statistically significant.

**Table 1:** Optimal classification results and performance comparisons in the three MI paradigms

Subjects	Dataset 1			Dataset 2			Dataset 3		
	MI tasks Parad			Two-class control Parad			Four-class control Parad		
	All Ch	C3, C4, Cz	Opt Ch	All Ch	C3, C4, Cz	Opt Ch	All Ch	C3, C4, Cz	Opt Ch
	Acc (%)	Acc (%)	Acc(%)	Acc (%)	Acc (%)	Acc (%)	Acc (%)	Acc (%)	Acc (%)
Sub1	67.8	88.9	92.8	95.6	92.7	99.3	75.5	69.4	87.1
Sub2	62.2	65.6	86.7	82.7	65.1	91.2	74.1	76.4	90.6
Sub3	62.2	61.7	82.8	85.9	51.8	94.1	76.3	72.7	83.3
Sub4	70.0	74.4	90.0	67.9	52.2	85.3	58.2	67.0	80.2
Sub5	71.1	79.4	90.0	93.5	76.2	96.1	63.4	63.4	77.6
Sub6	62.8	54.4	80.0	96.2	95.2	98.6	55.8	73.4	80.3
Sub7	64.4	69.4	80.0	92.2	93.8	98.1	77.7	71.2	81.8
Sub8	57.2	60.6	78.9	82.4	82.3	90.4	74.8	66.9	84.9
Mean	64.7	69.3	85.2	87.1	76.2	94.1	69.5	70.1	83.2
Std	4.6	11.2	5.4	9.5	18.0	4.9	8.8	4.2	4.2
*p* value	–	–	p = 7.0e−5	–	–	p = 0.024	–	–	p = 2.4e−4

In each dataset, the p-value is computed from one-way ANOVA among Opt Ch Acc, All Ch Acc and C3, C4, Cz Acc. It has to been noted that the subjects 1–8 in all the three datasets are not the same group of subjects

*Parad* paradigm, *Ch* channel, *Acc* accuracy, *Opt* optimal

Table 2 presents the numbers of optimal channels in all the subjects of the three datasets. The average numbers of optimal channels in each dataset show significant difference (p = 4.36e−4, one-way ANOVA), with the requirement of approximately 14, 22, and 29 channels respectively for MI tasks paradigm, two-class control paradigm, and four-class control paradigm. On average, compared to MI tasks paradigm, both the control paradigms required more channels to reach optimal accuracy. However, the difference between two control paradigms is not so obvious, although the average number of four-class control paradigm is bigger than that in two-class control paradigm. The number of optimal channels in Dataset3 has a relatively large fluctuation. The numbers of two subjects (Sub4 and Sub6) are close to the average number of 1-D cursor control, while the numbers of other subjects are far beyond that number. It seems that the numbers of optimal channels vary distinctively, and this is especially true when an experimental paradigm changes from an MI tasks paradigm to control paradigms. The significant difference among paradigms may be due to the intrinsic differences in experimental paradigms which may induce distinct EEG waves or EEG distributions over scalp.

**Table 2:** The numbers of Optimal Channels in all the subjects from the three MI Paradigms

Subjects	Sub1	Sub2	Sub3	Sub4	Sub5	Sub6	Sub7	Sub8	Mean	Std
Dataset1	11	11	12	16	19	5	20	18	14	5
Dataset2	35	19	16	20	19	20	22	24	22	6
Dataset3	32	30	43	18	34	23	27	25	29	8

### Accuracy over varying number of channels

**Fig. 3:**
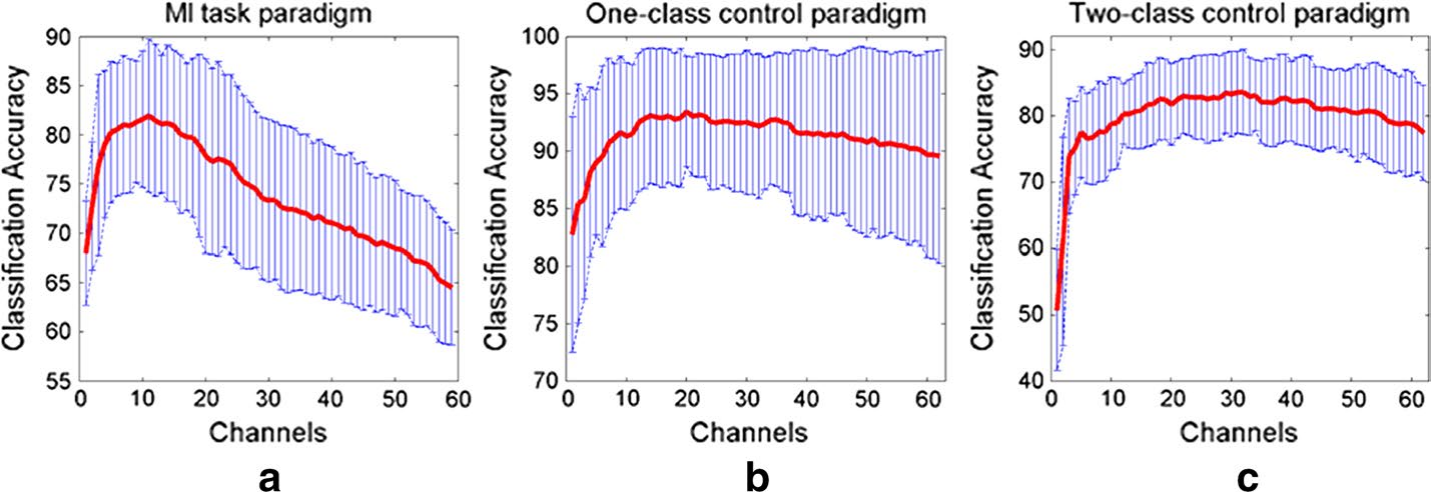
Overall accuracy curves showing the accuracy behaviors with the varying numbers of channels. For each subject, channel is added one by one in term of its channel weight. Channels with large weights have the priority of being added first. The overall accuracy curves are the average over eight subjects in each dataset. The *red line* is the mean accuracy curve and the *blue dot lines* represent the envelopes of ±standard deviation. **a** Average accuracy curve for MI tasks paradigm; **b** average accuracy curve for two-class control paradigm; **c** average accuracy curve for four-class control paradigm

In this section, the accuracy behaviors over varying number of channels are presented to show how the channel number impacts the classification performance in each MI paradigm. In each subject, channels are added one by one according to its channel weight until all the channels are used. Channels with larger weight have the priority to be added first. Overall accuracy behaviors averaged from all the subjects in each dataset are shown in Fig. 3. It is shown that for each paradigm, the accuracy reaches the optimal point at a certain point of channel number, and then degrades with the increase of used channel number. Compare subfigure (a) with subfigures (b) and (c), it is observed that the performances from MI tasks paradigm degrade more quickly and deeply along with the increase of channel number. Whereas, the accuracy curves from two-class control and four-class control are relatively stable.

### Distribution of optimal channels and frequency bands

**Fig. 4:**
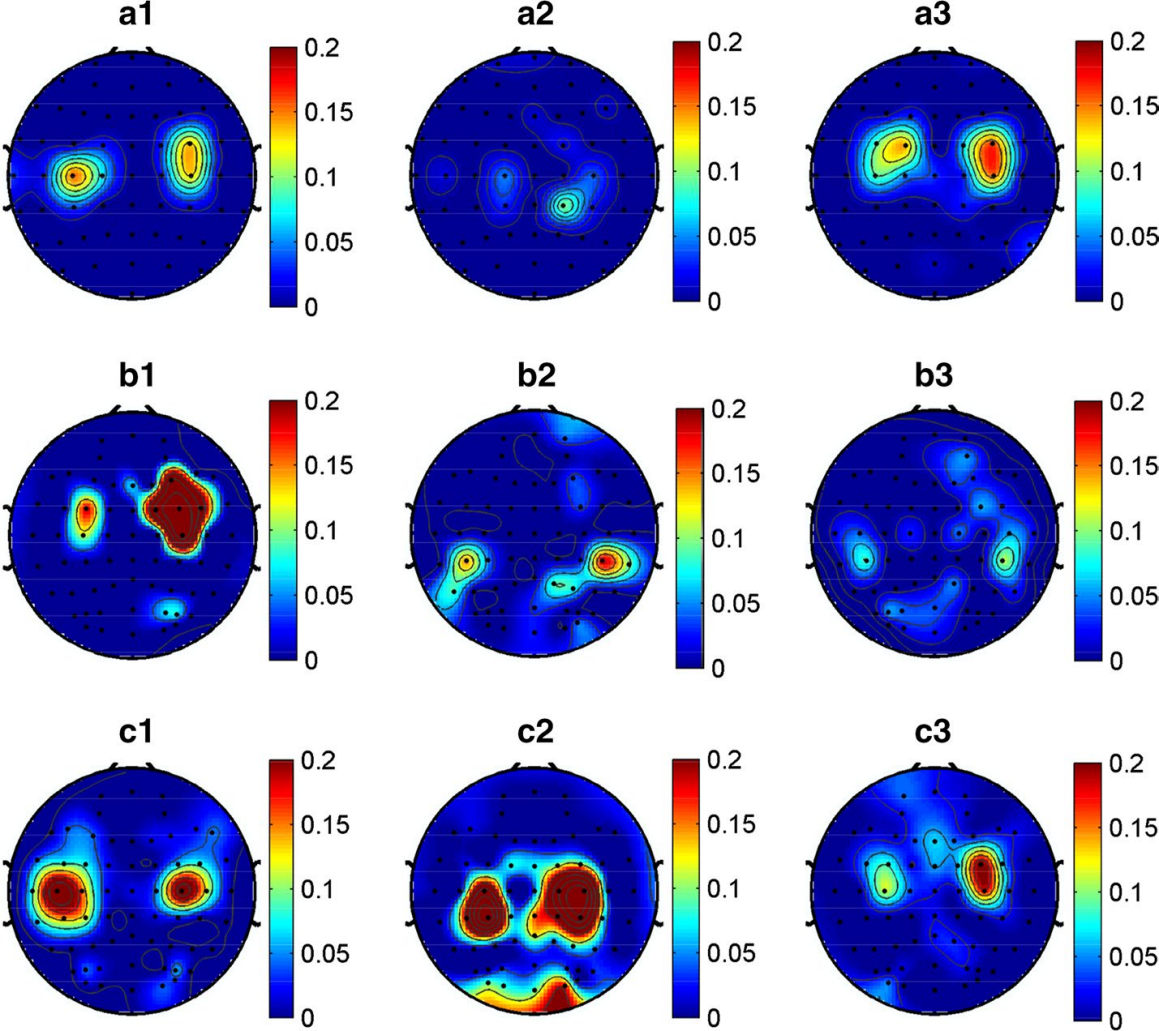
The optimal channel topographies of nine subjects. Topographies demonstrate which part of the cortex area the selected optimal channels locate over. The *color* indicates the importance of a channel in MI classification, and the importance of a channel is determined by its weight which is the sum of weights of the selected features. For non-optimal channel (discarded channel), its weight is set to zero here. **a1**, **a2**, **a3** represent three subjects from MI tasks paradigm, **b1**, **b2**, **b3** are from two-class control paradigm and **c1**, **c2**, **c3** are from four-class control paradigm

Used the method described in the “Methods” section, the optimal channels and their reserved frequency bands for each subject are determined. In this section, we are concerned about which part of brain regions these optimal channels locate over. So the selected optimal channels in each subject were mapped onto their corresponding locations in the electrode cap, and the weight of an optimal channel in topography is determined by its sum of reserved frequency band weights. For example, if 3 of 13 frequency bands in a channel are selected and the weights of the selected frequency bands are 0.001, 0.002 and 0.003 respectively, then the weight of this channel will be 0.006. As for non-optimal channels (discarded channels), their weights are set to zero. Figure 4 presents the optimal-channel topographies of 9 representative subjects from the three paradigms (3 for each paradigm).

It is shown in Fig. 4 that for the MI tasks paradigm, the optimal channels are mainly located over motor cortex with a good bilateral symmetry for each subject, and few channels scattered over other cortex regions. Whereas for both control paradigms, the spatial distributions of the optimal channels involves a broader brain area, centered in motor area. For two-class control paradigm, the distribution of each subject is much more scattered, especially for the latter two subjects (b2, b3), but the most important channels still locate over or near motor cortex, so is the case for the four-class control paradigm. This means that for control paradigms, though plenty of channels may contribute their impacts in MI classification, but the channels over motor area are still in a dominant position. Furthermore, the topographies of two-class control and four-class control paradigms show that there were active brain activities over visual cortex during motor imageries, particularly in four-class control paradigm. This may be induced by real-time visual feedback during the experiments. In comparison, the brain activities in MI tasks paradigm are much weaker.

**Fig. 5:**
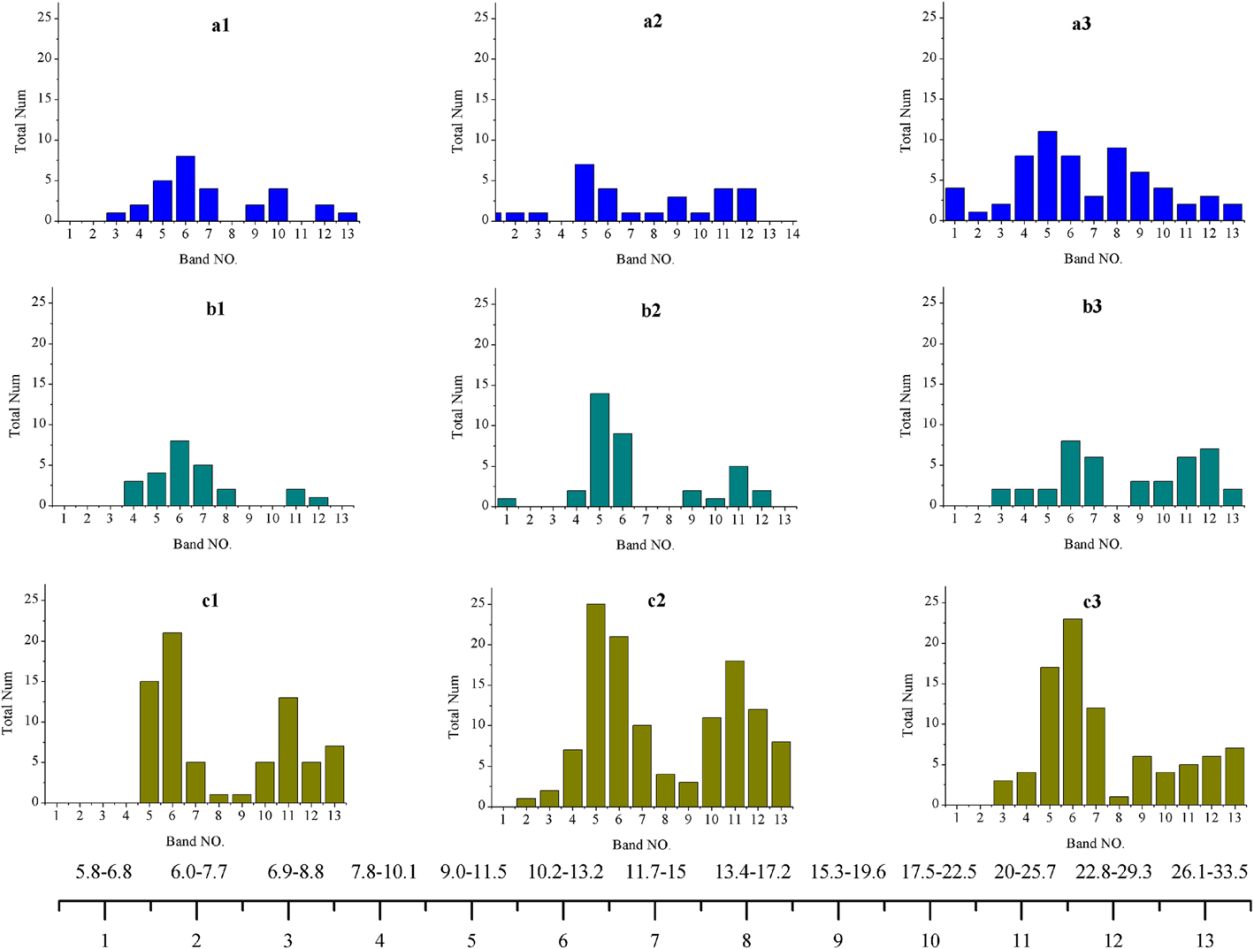
The usage of each frequency band in the representative subjects. The height of each *bar* (y axis) quantitatively reflects how many channels reserved this frequency band as an informative feature. The *numbers* in x axis represent the frequency band serial number. The frequency range corresponding to each band no. is shown at the *bottom* of the figure. The subjects are the same with the ones in Fig. 4. **a1**, **a2**, **a3** are the three subjects from MI tasks paradigm; **b1**, **b2**, **b3** from two-class control paradigm; **c1**, **c2**, **c3** from four-class control paradigm

**Fig. 6:**
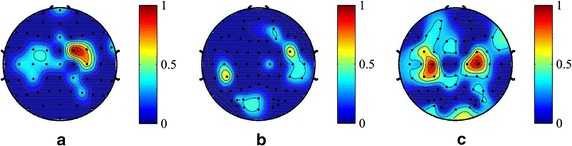
Averaged distributions of beta wave over three subjects in each paradigm. The beta wave (17–28 Hz) corresponds to the frequency band 10, 11, 12. The weight of each channel in the topographies is the average of channel weights from three representative subjects. **a** Averaged distribution for MI tasks paradigm; **b** averaged distribution for two-class control paradigm; **c** averaged distribution for four-class control paradigm

Figure 5 shows the usage conditions of 13 frequency bands in each representative subjects. The heights of 13 bars in each sub-figure quantitatively reflect how many channels out of the total channels (59 or 62) reserved this frequency band. It is shown that the usage of frequency bands varies across subjects, but several frequency bands are commonly used across the subjects and experimental paradigms. And those bands are bands No. 5–7 and bands No. 10–12, which agree with the frequency ranges of *mu* band (8–15 Hz) and *beta* band (17–28 Hz). *Mu* and *beta* bands are well known to be dominant during MI. Moreover, compared to the MI tasks paradigm and two-class control paradigm, the frequency bands 8–15 Hz and 17–28 Hz in four-class control paradigm are included in more channels. The spatial locations of beta wave are presented in Fig. 6, where the distribution of beta wave is averaged from the three representative subjects in each paradigm. It is shown that the beta wave mainly locates over motor cortex and its distribution in four-class control paradigm is broader than the other two paradigms.

### Comparison with other feature selection methods

**Fig. 7:**
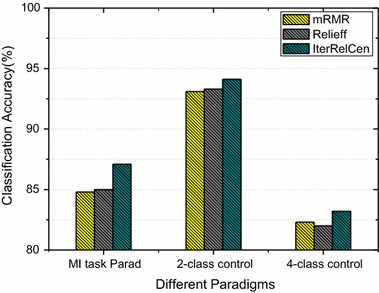
Performance comparison among different channel selection methods. *Yellow bars* represent for mRMR, *gray bars* for Relief/Relieff and *dark blue bars* for IterRelCen

The algorithm mRMR is effective in feature selection and has been proved to outperform plenty of feature selection methods, such as MaxDep and MI (mutual information) through extensive experiment comparisons [41, 42]. So in the present study, mRMR was also adopted for channels selection and accuracy calculation for the purpose of comparison. Relief/Relieff was also used for comparison, since our proposed method is based on Relief/Relieff. The results in Fig. 7 show that compared to mRMR and Relief/Relieff, the method IterRelCen performed better, achieving the best average accuracies over all the three datasets. This means that IterRelCen has a relatively strong ability to select better channel and features.

## Discussions

### An enhanced method

In a sensorimotor rhythm based BCI system utilizing MI, the ability to effectively classify distinct patterns of MI is crucial. It was discovered that the use of multiple channels improves classification performance. However, when a large number of channels are used, it is better to conduct channel selection to eliminate noisy and redundant channels. The obtained results proved that our proposed method based on IterRelCen performed excellently in classification and optimal channel selection. The advantages of our method are mainly embodied in two aspects. First, the underlying information relevant with motor imageries in single channel is completely explored by dividing channel signal into 13 overlapping frequency bands, avoiding the possibility of rejecting useful channels. Second, an enhanced method based on Relief/Relieff strengthens the ability of optimal feature selection. Compared to Relief/Relieff, IterRelCen solved the noise sensitive problem, making itself much stronger and robust in noisy dataset analysis. Using iterative deletion strategy to eliminate the influence of noisy features in nearest neighbor search, the weights arrived from these sample data could reflect the importance of features more accurately. In the present study, by combining IterRelCen algorithm with this feature extraction method, comprehensive information without irrelevant features was flexibly extracted. The adoption of our proposed method may also account for the results that in two-class and four-class control paradigms, the numbers of optimal channels are relatively large. That is because of the fine division of frequency bands combined with flexible selection, and it will not let any informative frequency band in any channel be ignored, whereas such missing or ignoring may happen to other methods.

### Number of related channels in different MI paradigms

Our results suggested that in the three MI paradigms, the channel number seems to impact in optimizing the performance of MI classification. The obtained results reveal that the average numbers of optimal channels were significantly different among the three MI paradigms. From MI task paradigm to two-class control paradigm, and to four-class control paradigm, the number of required channels for optimizing the classification accuracy increased. This is particular true when a paradigm changes from MI tasks paradigm to any one of the control paradigms. However, one limitation of this study is that this conclusion is only drawn from a small number of subjects. And this result needs to be further confirmed through data analysis over a large number of subjects.

A reasonable explanation for this conclusion is that the intensity of involved brain activities and the region of involved brain areas vary in different MI experimental paradigms. Research indicates that an increase of task complexity or attention results in an increased magnitude of brain activities [28, 43]. From this perspective, the MI task paradigm is the simplest one, where subjects only perform imagination of left or right hand movement. However, for the control paradigms, their complexity of experimental paradigms are reflected in two aspects: one is that they need to concentrate more on mental tasks in order to adjust the control strategy and brain states in real time; the other is that the control paradigms provide visual stimulation which is not included in the MI tasks paradigm. Another explanation accounts for the conclusion is suggested in [10], that visual feedback during MI could augment brain activity over the motor areas. Thus, augmented brain activity may expand to a broader brain region, and therefore it is reasonable that the number of relevant channels is larger in two-class and four-class control paradigms than the MI tasks paradigm. The difference between any one of the control paradigms and MI tasks paradigm is significant. As to the distinction between two control paradigms, the four-class control paradigm demands more patterns of brain states than the two-class control paradigm in order to generate four distinct mental tasks. Furthermore, switching of more brain states may result in a heavier burden of brain cognition and more focused attention, which may induce stronger brain activities in a broader brain area than the two-class control paradigm. So compared to two-class control, it was found that most of subjects in the four-class control paradigm correlates with more channels. However, there are also subjects whose numbers of optimal channels are close to the average level of the two-class control paradigm, such as Sub4 and Sub6 shown in Table 2. This can be explained by this: for some well-training subjects, the induced EEGs are more focused in cortex and the switching of brain states is not a burden for them. Such subjects may induce MI related EEG in a relatively small range, similar with the two-class control paradigm. And thus the numbers of optimal channels may be close to the two-class control paradigm.

A minor drawback of our study design is that the experimental conditions are not strictly controlled due to the limitation of experimental condition: first, the three datasets were not strictly collected from the same group of subjects; second, there is a minor difference in electrode setups because of variation in experimental conditions between datasets.

### Relevant channels and frequency bands

We can observe the locations of relevant channels from the representative subjects in Fig. 4. It is shown that the most important channels were mainly over or near left or right sensorimotor areas with an almost symmetrical distribution in most subjects. This result is consistent with the findings from previous studies [26, 43], which show that motor imagery induces brain activity in primary motor area, supplementary motor area, etc.

As shown in Fig. 5, frequency bands that play the most important role in classification are bands 5–7 and bands 10–12, corresponding to 8–15 Hz and 17–28 Hz respectively. These bands have a large overlap with the *mu* band (8–12 Hz) and *beta* band (18–26 Hz). This finding is consistent with the existing literature which has already shown that the phenomenon of ERS or ERD is notably observed in *mu* and *beta* bands [12, 34]. Though the *mu* and *beta* frequency bands are dominant in classification, the other bands also contribute to the improvement of classification accuracy. From this perspective, it is wise to divide a single-channel signal into multiple frequency-domain signals, avoiding loss of partial related information by rejecting a channel or introduction of irrelevant information by adopting a whole channel signal. So in such condition, the combination of frequency decomposition with IterRelCen algorithm could provide a useful approach for selection of related features.

Furthermore, it is beneficial to have a clear understanding of which channels and bands are of most importance, since this could provide a quick reference on utilization of the most important channels and frequency bands under the situation where it is required to use the least number of channels with acceptable classification accuracy.

### Limitations and future work

We performed an offline analysis on three datasets recorded from three different types of online experiments in this study. Since the offline and online classifications have distinct characteristics, a further test in a real online experimental environment should be conducted to confirm the present findings. In addition, the number of subjects is relatively small, and the obtained results need to be further confirmed over a large number of subjects. Furthermore, the classifier used in the present study is time-consuming, and it is not suitable for real-time MI classification due to its high demand on computational speed. Thus it is necessary to develop an efficient algorithm for multi-class classification meeting the requirements of online modality.

## Conclusions

In summary, we have developed a novel channel selection method IterRelCen and applied it to three different datasets, respectively collected from a MI tasks paradigm, a two-class control paradigm, and a four-class control paradigm. The classification accuracies show that the proposed approach is capable of effectively classifying multi-class motor imagery patterns and it outperformed several widely used feature selection methods. The channels and feature bands that are well known to be important (from a neurophysiological view) are consistent with the results obtained by our study.

An interesting finding in this study is that the obtained results about channel selection reveal that there exists a distinct difference of the number of optimal channels in the three MI based BCI paradigms. From a MI task paradigm, to a two-class control paradigm, and to a four-class control paradigm, the required channels for optimizing the classification accuracy increased. This conclusion is particularly true when a paradigm changes from MI tasks paradigm to any one of the control paradigms. The reasons for this may be attributed to the following two aspects: (1) increased task complexity or attention results in an increased magnitude of brain activities; (2) real-time visual feedback during MI could augment brain activity. The present results could provide useful information for channel selection in real MI based BCI experiments.

## Abbreviations

BCI: brain computer interface
EEG: scalp-recorded electroencephalogram
MI: motor imagery
ERD: event-related desynchronization
ERS: event-related synchronization
SVM: support vector machine
RBF: radial basis function
mRMR: minimal redundancy maximal relevance
